# Generation, Annotation, and Analysis of a Large-Scale Expressed Sequence Tag Library from *Arabidopsis pumila* to Explore Salt-Responsive Genes

**DOI:** 10.3389/fpls.2017.00955

**Published:** 2017-06-07

**Authors:** Xianzhong Huang, Lifei Yang, Yuhuan Jin, Jun Lin, Fang Liu

**Affiliations:** Special Plant Genomics Laboratory, College of Life Sciences, Shihezi UniversityShihezi, China

**Keywords:** *Arabidopsis pumila*, *Olimarabidopsis pumila*, ephemerals, salt stress, EST, gene expression, salt shock

## Abstract

*Arabidopsis pumila* is an ephemeral plant, and a close relative of the model plant *Arabidopsis thaliana*, but it possesses higher photosynthetic efficiency, higher propagation rate, and higher salinity tolerance compared to those *A. thaliana*, thus providing a candidate plant system for gene mining for environmental adaption and salt tolerance. However, *A. pumila* is an under-explored resource for understanding the genetic mechanisms underlying abiotic stress adaptation. To improve our understanding of the molecular and genetic mechanisms of salt stress adaptation, more than 19,900 clones randomly selected from a cDNA library constructed previously from leaf tissue exposed to high-salinity shock were sequenced. A total of 16,014 high-quality expressed sequence tags (ESTs) were generated, which have been deposited in the dbEST GenBank under accession numbers JZ932319 to JZ948332. Clustering and assembly of these ESTs resulted in the identification of 8,835 unique sequences, consisting of 2,469 contigs and 6,366 singletons. The blastx results revealed 8,011 unigenes with significant similarity to known genes, while only 425 unigenes remained uncharacterized. Functional classification demonstrated an abundance of unigenes involved in binding, catalytic, structural or transporter activities, and in pathways of energy, carbohydrate, amino acid, or lipid metabolism. At least seven main classes of genes were related to salt-tolerance among the 8,835 unigenes. Many previously reported salt tolerance genes were also manifested in this library, for example *VP1, H^+^-ATPase, NHX1, SOS2, SOS3, NAC, MYB, ERF, LEA, P5CS1*. In addition, 251 transcription factors were identified from the library, classified into 42 families. Lastly, changes in expression of the 12 most abundant unigenes, 12 transcription factor genes, and 19 stress-related genes in the first 24 h of exposure to high-salinity stress conditions were monitored by qRT-PCR. The large-scale EST library obtained in this study provides first-hand information on gene sequences expressed in young leaves of *A. pumila* exposed to salt shock. The rapid discovery of known or unknown genes related to salinity stress response in *A. pumila* will facilitate the understanding of complex adaptive mechanisms for ephemerals.

## Introduction

Salinity is a serious problem worldwide, with more than 800 million hectares of land across the world estimated to be affected by high salinity (Munns, 2005). Furthermore, soil salinization has increased in recent years as a result of anthropogenic actions such as irrigation.

As sessile organisms, plants are subjected to various environmental stresses, such as drought, salinity, and temperature extremes, during their life cycle. Soil salinity is one of the major abiotic factors constraining plant growth and crop production. About 100 million hectares of land in China is exposed to primary salinity or secondary salinity, accounting for approximately 10% of the world’s saline and alkaline land resources. As the largest province in northwest China, Xinjiang occupies one-sixth of the land of China; however, more than one-third of the land in Xinjiang suffers from primary of secondary salinization (Zhao et al., 2013). Therefore, achieving a greater understanding of the genetics of salt tolerance in plants and its exploitation in improving salt tolerance of plants, especially crops, is one of the most important goals in China.

Salinity affects plant growth and development severely, causing crop production loss worldwide (Munns, 2002). Salinity stress results in changes in various physiological and metabolic processes that limiting plant growth and productivity. Osmotic stress and ion toxicity are currently considered to be the two major components of the plant-salt stress response (Gupta and Huang, 2014). First, high salinity causes osmotic stress by reducing water uptake; in addition, it results in ion toxicity due to the absorption of large numbers of Na^+^ and Cl^-^ ions through the plant root system (Munns, 2002, 2005). Furthermore, high salinity can interfere with various physiological and metabolic processes of plants, such as photosynthesis, essential ion uptake, cell membrane integrity, nutrient balance and detoxification of reactive oxygen species (ROS) (Munns and Tester, 2008; Rahnama et al., 2010).

In order to survive in high-salt soils, plants have evolved various physiological and biochemical mechanisms to protect themselves from salinity damage. The principal salt tolerance mechanisms include ion homeostasis involving Na^+^/K^+^ exclusion and compartmentalization, biosynthesis of osmoprotectants and compatible solutes, activation of antioxidant enzymes and synthesis of antioxidant compounds, and hormone modulation (Asada, 1999; Hasegawa et al., 2000; Peleg and Blumwald, 2011). Although much progress has been made in our understanding the genetic bases of salinity tolerance in plant, a lot of questions remain. More plants that are salt-responsive or salt-tolerant should be taken into account as research materials, rather than merely focusing on the widely used model plant *Arabidopsis thaliana*. Naturally occurring variability of species related to *A. thaliana* may be a valuable source for physiological, biochemical, and genetic analyses (Alonso-Blanco and Koornneef, 2000; Koornneef et al., 2004), which can provide new insights into the response mechanisms of plants to environmental stresses.

Owing to the extreme ecological environment, salt-tolerant or salt-adapted organisms are abundant in the salinized and semi-salinized land of Xinjiang. *Arabidopsis pumila* (syn. *Olimarabidopsis pumila*), a brassicaceous spring ephemeral plant, is closely related to the model plant *A*. *thaliana*, but is adapted to more stressful environments, and it has a high propagation rate (Hoffmann et al., 2010). Because of long-term adaptation to harsh environments, *A. pumila* has evolved enhanced activities of photosystem II under high light conditions (Tu et al., 2016). Recently, we found that *A. pumila* is distributed widely in the extreme environments of Xinjiang, showing considerable phenotypic variation. An extremophyte, *A. pumila* is adapted not only to the arid climate but is also more tolerant to salinity stress than is *A. thaliana*, thus representing an ideal plant system for genes mining for salt tolerance (Xu et al., 2013; Zhao et al., 2013).

Expressed sequence tags (ESTs), which are short, single-pass sequence reads from cDNA, have proven to be an efficient and rapid strategy to identify novel genes involved in tolerance to environmental stress. Large-scale cDNA sequencing and EST analyses have been successfully used to identify genes that may be related to stress tolerance in a number of salinity-adapted species such as *Suaeda salsa* (Zhang et al., 2001), *Avicennia marina* (Mehta et al., 2005), *Thellungiella halophila* (Taji et al., 2008), *Salicornia brachiata* (Jha et al., 2009), *Suaeda asparagoides* (Ayarpadikannan et al., 2012), and *Gossypium barbadense* (Zhou et al., 2016).

To obtain mRNA transcriptional profiles and to understand molecular adaptation mechanisms in response to salt stress, a high-quality normalized cDNA library was constructed from young leaves of *A. pumila* plants exposed to 500 mM NaCl shock for 14 h by using the gateway technology (Zhao et al., 2013). A total of 894 ESTs were generated by sequencing analysis, which were assembled into 736 unique sequences consisting of 72 contigs and 664 singletons. All the unigenes were categorized based on Gene Ontology (GO) and on preliminary analysis of the potential roles of the ESTs in response to salt stress.

However, the small number of ESTs could not provide a complete mRNA transcriptional profile. In this study, a more comprehensive EST library was generated by means of random sequencing of clones from this cDNA library, followed by functional characterization of putative genes and identification of genes exhibiting differential expression under salt shock conditions by quantitative real-time PCR (qRT-PCR). The EST database obtained will provide new insights into salt-adaptive mechanisms of *A. pumila* and will be also an important resource for comparative genomics studies among brassicaceous species.

## Materials and Methods

### Plant Material and Salt Stress Treatment

*Arabidopsis pumila* seeds were surface-sterilized by soaking in 70% (v/v) ethanol for 2 min, and then in 2.8% (w/v) sodium hypochlorite solution containing 0.1% surfactant (Triton X-100; Sigma-Aldrich, Munich, Germany) for 30 min, and finally rinsed five times with sterile distilled water. Seeds were stratified for 3 days at 4°C in darkness to synchronize germination, and then plated on Petri dishes with half-strength Murashige-Skoog (MS) salt mixture (pH 5.7; Duchefa, Haarlem, Netherlands), 1% (w/v) sucrose, and 0.8% (w/v) agar. Petri dishes were then placed in a illumination incubator at 22°C under long-day (LD) conditions (16 h light/8 h dark). After 7 days, the seedlings were transplanted into pots containing peat soil and vermiculite (1:1) and kept in a plant growth chamber with a 16-h photoperiod, and the light intensity for *A pumila* growth is 200 μmol m^-2^ s^-1^. To examine gene expression during the salt shock, The 4-week-old plants were watered with 0.5 × MS nutrient solution supplemented with 500 mM NaCl as described previously (Zhao et al., 2013). To validate the candidate genes associated with stress related ESTs, qRT-PCR was performed to detect their expression patterns in response to salt stress at different time. Leaves of the treated plants were separately harvested at 0, 0.5, 3, 9, 14, or 24 h time points of salt shock, respectively, and all the samples were immediately frozen in liquid nitrogen and stored at -80°C for gene expression analyses. Each treatment was conducted in three biological replicates.

### EST Sequencing, Editing, and Assembly

The phenotype evaluation assay revealed preliminary that the plantlet of *A. pumila* began to appear wilting in 1-month-old seedling shocked by 500 mM NaCl for 16–18 h. The stages before 16 h salt treat may be more important as gene expression usually occurs before morphological changes. The normalized and full-length cDNA library of *A. pumila* young leaves shocked by 500 mM NaCl for 14 h were constructed by Zhao et al. (2013). Clones from this library were randomly selected from Luria-Bertani (LB) agar plates supplemented with 50 μg/ml kanamycin. Cultivated in 37°C overnighted in standard LB/kanamycin medium, plasmids were isolated from randomly selected clones. Sequencing was carried out from the 5′ end of the cDNA inserts with the M13 forward primer (5′-GTAAAACGACGGCCAG-3′) using an ABI PRISM 3730xl automated DNA sequencer (Applied Biosystems, Grand Island, NY, United States) at the Sequencing Center of the Beijing Genomics Institute (Beijing, China).

All sequences were cleaned by removing the low-quality sequences, such as the chimeric clones, the contaminating sequences, the repeat sequences, and poly(A) tails. Then the vector sequences were eliminated using VecScreen program^1^. After cleaning, sequences shorter than 100 nucleotides were discarded. Finally, high-quality ESTs were aligned and assembled into contigs and singletons using Codon Code Aligner software^2^ with 98% sequence identity and a 40-bp minimum match length. The contigs and singletons should thus correspond to sequences of unique genes (unigenes).

### Unigene Function Annotation and Classification

To get their putative functional information, all the unigenes were annotated. First, all the unique sequences were searched for putative open reading frames (ORF) with the program ORFfinder^3^, and the largest ORF sequences were used for functional analysis. All the unigenes were then aligned to protein databases like NCBI non-redundant protein (Nr) database, Protein Sequence Database (Swiss-Prot)^4^, and Cluster of Orthologous Groups of Proteins (COG)^5^ by blastx (*E*-value ≤ 10^-5^), and the NCBI non-redundant nucleotide database (Nt) by blastn (*E*-value ≤ 10^-5^).

In addition, Blast2GO (Conesa et al., 2005) program were used to get GO annotation of unigenes for describing cellular component (CC), molecular function (MF), and biological process (BP) with Nr annotation. Finally, the analysis of high-level functions and utilities of biological system were also carried out with the Kyoto Encyclopedia of Genes and Genomes (KEGG) Automatic Annotation Server (KAAS) using the single-directional best hit (SBH) method to assign orthologs^6^ (Moriya et al., 2007).

### Identification of Putative Transcription Factors

Unigenes were aligned to a plant transcription factor database (PlantTFDB4.0)^7^ by blastx (*E*-value ≤ 10^-5^) to identify putative transcription factors (Jin et al., 2017).

### qRT-PCR Confirmation

Total RNA were extracted using RNeasy Plant Mini Kit (Qiagen, GmbH, Germany) and treated with RNase-free DNase (Qiagen) following the manufacturer’s instructions. The quality and quantity of total RNA were monitored using ND-1000 spectrophotometer (NanoDrop Technologies, Wilmington, DE, United States).

The cDNA synthesis reactions were performed using the Superscript First-Strand Synthesis System (Invitrogen, Carlsbad, CA, United States) according to the manufacturer’s instructions. Gene-specific primers for qRT-PCR analyses (**Supplementary File S1**) were designed using Primer Premier 6.0 software (Premier Biosoft International, Palo Alto, CA, United States). qRT-PCR was performed on an Applied Biosystems 7500 Fast Real-Time PCR System (Life Technology, Foster City, CA, United States) in a 25-μl volume containing 10 ng of cDNA, 5 pM of each primer, and 25 μl of Fast SYBR Green Master Mixture (CWBIO, Beijing, China) according to the manufacturer’s protocol. Primers of Actin2-F (5′-CACCGTGAGTGGCAAAGAAGGGA-3′) and Actin2-R (5′-AACGACCTTAATCTTCATGCTGC-3′) were used to amplify *Actin2* gene (Xu et al., 2013), which was used as an internal control. Three replicate assays were performed with independently isolated RNA; each RT reaction was loaded in triplicate. Relative expression levels of the selected unigenes were presented using the 2^-ΔΔCt^ method (Livak and Schmittgen, 2001).

## Results

### ESTs Sequencing and Assembly

**FIGURE 1:**
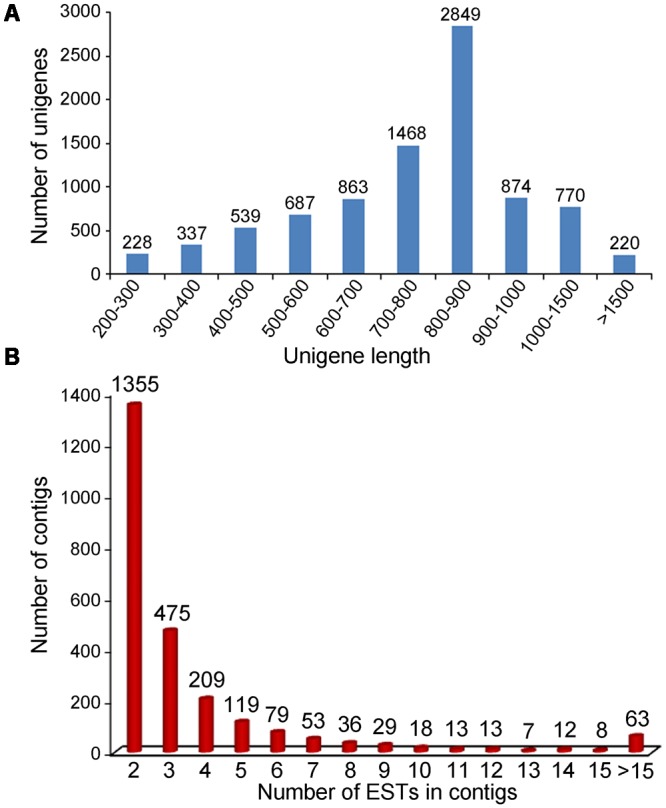
Characterization of the ESTs and contigs. **(A)** Sequence length distribution of the ESTs after assembly; **(B)** Distribution of 2,346 contigs based on the number of clustered ESTs.

A total of 19,923 cDNA clones randomly selected were successfully single-pass sequenced from the 5′ terminal, generating 16,014 high-quality ESTs (80.4%) with an average length of 546 bp after cleaning. All of these ESTs sequences have been deposited in GenBank with accession number of JZ932319 ∼ JZ948332. The sequence length distribution of high-quality EST sequences in the clusters is shown in **Figure 1A**. The clean ESTs were assembled into 8,835 unigenes, including 2,469 (27.9%) contigs and 6,366 (72.1%) singletons. The average length of the unique sequences was 794 bp, ranged from 200 to 2,610 bp. The detailed information of these ESTs was summarized in **Table 1**.

**Table 1:** EST sequence and assembly statistics.

Feature	Value
Total number of sequence reads	19,923
High-quality sequences	16,014
Number of contigs	2,469
Number of singletons	6,366
Number of ESTs in contigs	9,648
Number of unique sequences	8,835
Novelty (%)	55.2
Redundancy (%)	44.8
Average length of unigene sequences (bp)	794
Average length of contigs (bp)	1004
Average length of singletons (bp)	713
Longest length of unique sequences (bp)	2,610

Expressed sequence tags per contig averaged at 3.9 in number, while the highest number reached 72. The distribution of EST frequencies after clustering was shown in **Figure 1B**. Of the 2,469 contigs, 1,335 (54.1%) contained 2 ESTs, 475 (19.2%) contained 3 ESTs, 209 (8.5%) contained 4 ESTs, 119 (4.8%) contained 5 ESTs, and the remaining 13.4% contained 6 or more ESTs. The results showed that the redundancy in the normalized library was relatively low, confirming that the cDNA library was normalized well for EST generation.

The clusters that contain more than 30 ESTs represent the most abundant unigenes and their annotations were summarized in the **Table 2** and **Supplementary File S2**. The abundant transcripts associated with photosynthesis were obtained such as chlorophyll *a*/*b* binding protein (CAB) (Contig5032, Contig5031, Contig5022, Contig5017, including 228 ESTs), light-harvesting complex II chlorophyll *a*/*b* binding protein (LHC) (Contig5030, Contig5026, Contig5011, including 152 ESTs), ribulose bisphosphate carboxylase (RuBisCO) small chain (RBCS) (Contig5029, Contig5028, including 119 ESTs) (Jha et al., 2009), suggesting photosynthesis is still active under NaCl treatment. Genes related to defense were also expressed abundantly including ribulose activase (RCA) (Contig5021, Contig5019, including 83 ESTs), glutathione *S*-transferase 16 (GSTF3) (Contig5015, Contig5012, including 67 ESTs) involved in defense against reactive oxidative species, metallothionein-like protein (MT1C) (Contig5014, including 39 ESTs) involved in response to copper ion (Hall, 2002), catalase 2 involved in response to oxidative stress (CAT3) (Contig5024, Contig5025, including 105 ESTs), translationally controlled tumor protein (TCTP) (Contig5013, including 33 ESTs) involved in auxin homeostasis, cysteine proteinase RD19a (Coting5007, including 30 ESTs) involved in response to salt stress (Hasegawa et al., 2000; Gupta and Huang, 2014). Similarly a large number of these genes were also reported in the ESTs analyses of other plant species (Zhang et al., 2001; Kore-eda et al., 2004; Jha et al., 2009; Li et al., 2014).

**Table 2:** The most abundant ESTs detected in *A. pumila* leaf cDNA library.

Unigene name	ESTs number	Annotation	Source organism	*E*-value
Contig5032	72	Chlorophyll *a*/*b* binding protein 1	*Arabidopsis thaliana*	1.00*E* - 151
Contig5031	69	Chlorophyll *a*/*b* binding protein 1	*Arabidopsis thaliana*	1.00*E* - 150
Contig5033	69	Polyubiquitin 10	*Arabidopsis thaliana*	0
Contig5030	68	Light-harvesting complex II chlorophyll *a*/*b* binding protein 1	*Arabidopsis thaliana*	1.00*E* - 152
Contig5029	62	Ribulose bisphosphate carboxylase (RuBisCO) small chain 1A	*Arabidopsis thaliana*	4.00*E* - 95
Contig5028	57	RuBisCO small chain 3B	*Arabidopsis thaliana*	2.00*E* - 89
Contig5025	53	Catalase 2	*Brassica rapa* subsp.	0
Contig5026	53	Light-harvesting complex II chlorophyll *a*/*b* binding protein 1	*Arabidopsis thaliana*	1.00*E* - 152
Contig5024	52	Catalase 2	*Brassica rapa* subsp.	0
Contig5022	49	Chlorophyll *a*/*b* binding protein 151	*Arabidopsis thaliana*	1.00*E* - 152
Contig5021	44	RuBisCO/oxygenase activase	*Arabidopsis lyrata* subsp.	0
Contig5014	39	putative metallothionein protein type 1	*Brassica napus*	3.00*E* - 22
Contig5019	39	RuBisCO/oxygenase activase	*Arabidopsis lyrata* subsp.	0
Contig5016	38	Polyubiquitin 10 (UBQ10) mRNA	*Arabidopsis thaliana*	0
Contig5017	38	Chlorophyll *a*/*b* binding protein 2/3	*Arabidopsis thaliana*	1.00*E* - 150
Contig5015	37	Glutathione *S*-transferase 16	*Arabidopsis thaliana*	1.00*E* - 111
Contig5005	33	Photosystem II subunit R	*Arabidopsis thaliana*	1.00*E* - 114
Contig5013	33	Translationally controlled tumor protein	*Thellungiella halophila*	3.00*E* - 86
Contig5012	31	Glutathione *S*-transferase 16	*Arabidopsis thaliana*	0
Contig5011	31	Light-harvesting complex I chlorophyll *a*/*b* binding protein 3	*Arabidopsis thaliana*	1.00*E* - 146
Contig5012	31	Glutathione *S*-transferase 16	*Arabidopsis thaliana*	1.00*E* - 111
Contig5007	30	Cysteine proteinase RD19a	*Arabidopsis thaliana*	0

*The *E*-value is the expert value which describes the random background noise. The lower the *E*-value, or the closer to zero, the more “significant” the match is.*

**FIGURE 2:**
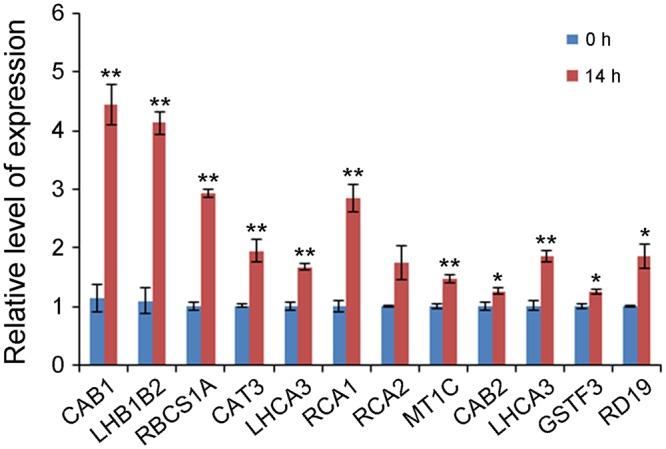
Gene expression levels of 12 selected enriched unigenes under 0 and 14 h NaCl stress conditions by qRT-PCR. *Y*-axis represents gene expression relative to *A. pumila Actin 2* (JZ151991). Significant differences at ^∗∗^*P* < 0.01 and ^∗^*P* < 0.05, respectively, according to Student’s *t*-test compared to wild type or mutant, respectively. Data represent the mean ± SE obtained from three biological replicates.

To determine whether the putative enriched genes were highly expressed, their expression levels were detected under control and 500 mM NaCl shock conditions by qRT-PCR. As shown in **Figure 2**, of the selected 12 genes, the transcriptions of 11 genes, including *CAB1* (Contig5032), *LHB1B2* (Contig5030), *RBCS1A* (Contig5029), *CAT3* (Contig5024), *LHCA3* (Contig5022), *RCA1* (Contig5021), *MT1C* (Contig5014), *CAB2* (Contig5017), *LHCA3* (Contig5011), *GSTF3* (Contig5012), and *RD19* (Contig5007), were truly abundant and significantly up-regulated at 14 h of treatment.

The prediction of ORFs revealed a high amount of putative proteins. Of 8,835 unique sequences, 8,585 (97.2%) were able to detect ORFs that were longer than 100 bp, with the longest ORF of 2,265 bp (**Supplementary File S3**), and the average ORF was 558 bp. The translated peptide sequences had a length ranging from 33 to 754 amino acids, with an average size of 185 amino acids.

### Unigene Functional Annotation and Categorization

**FIGURE 3:**
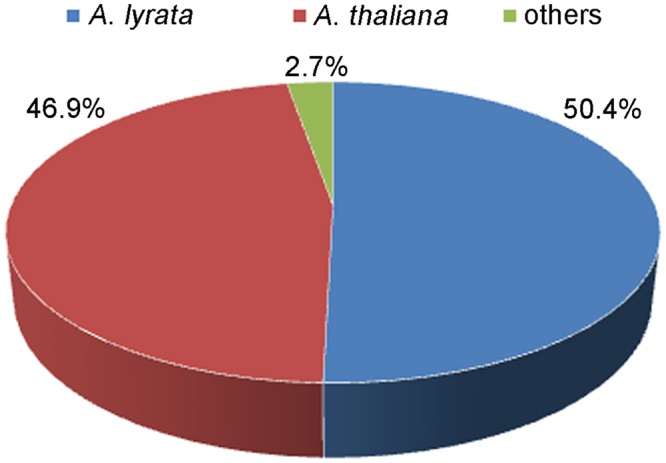
The species distribution of the unigenes in non-redundant (Nr) protein database.

The blastx search revealed that there were 8,436 (95.5%) unigenes showing significant similarity to proteins in NCBI Nr database. Of the 8,436 unigenes, 8,011 (95%) showed similarities to proteins of known function, and only 425 (5%) showed similarities to predicted proteins of unknown function. In species distribution analysis, 4,255 (50.4%) and 3,960 (46.9%) sequences had best blastx hit with *Arabidopsis lyrata* and *A. thaliana* database, respectively, while only 221 (2.7%) of annotated sequences had similarity with other plant species (**Figure 3**).

### COG Function Classification

Because the COG database is assumed to be an useful platform for genome-scale analyses for functional annotation of newly sequenced genomes and evolution from the macro (Tatusov et al., 2000), we then mapped all the annotated unigenes to the COG database to explore the distribution of gene function of this species. We found that 3,160 (35.8%) unigenes with COG annotation could be grouped into 24 COG categories (**Table 3**). The “general function prediction only” category represented the largest group (557 unigenes, 17.6%), followed by the categories of “posttranslational modification, protein turnover, chaperones” (409 unigenes, 12.9%), “translation, ribosomal structure and biogenesis” (385, 12.2%), “energy production and conversion” (289 unigenes, 9.1%), “carbohydrate transport and metabolism” (247 unigenes, 7.8%), “amino acid transport and metabolism” (209 unigenes, 6.6%), “lipid transport and metabolism” (145 unigenes, 4.6%) and “inorganic ion transport and metabolism” (120 unigenes, 3.8%). In addition, 79 (2.5%) unigenes were assigned to the category of “function unknown.” Whereas, only three sequences were assigned into “cell motility,” and one were assigned into “nuclear structure” category, respectively. A number of functional annotations in these 24 COG categories, for example, “energy production and conversion,” “inorganic ion transport and metabolism,” “signal transduction mechanisms,” and “defense mechanisms” are closely related to salt stress.

**Table 3:** COG function classification of all unigenes.

Class definition	Number	Percent^∗^
General function prediction only	557	17.6%
Posttranslational modification, protein turnover, chaperones	409	12.9%
Translation, ribosomal structure, and biogenesis	385	12.2%
Energy production and conversion	289	9.1%
Carbohydrate transport and metabolism	247	7.8%
Amino acid transport and metabolism	209	6.6%
Lipid transport and metabolism	145	4.6%
Inorganic ion transport and metabolism	120	3.8%
Signal transduction mechanisms	105	3.3%
Cell wall/membrane/envelope biogenesis	99	3.1%
Transcription	77	2.4%
Coenzyme transport and metabolism	82	2.6%
Function unknown	79	2.5%
Secondary metabolites biosynthesis, transport, and catabolism	73	2.3%
Replication, recombination, and repair	61	1.9%
Intracellular trafficking, secretion, and vesicular transport	53	1.7%
Nucleotide transport and metabolism	46	1.5%
Defense mechanisms	36	1.14%
Chromatin structure and dynamics	32	1.01%
Cytoskeleton	30	0.95%
RNA processing and modification	12	0.38%
Cell cycle control, cell division, chromosome partitioning	10	0.32%
Cell motility	3	0.09%
Nuclear structure	1	0.03%

*^∗^Percent is No./3,160.*

### GO Annotation

**FIGURE 4:**
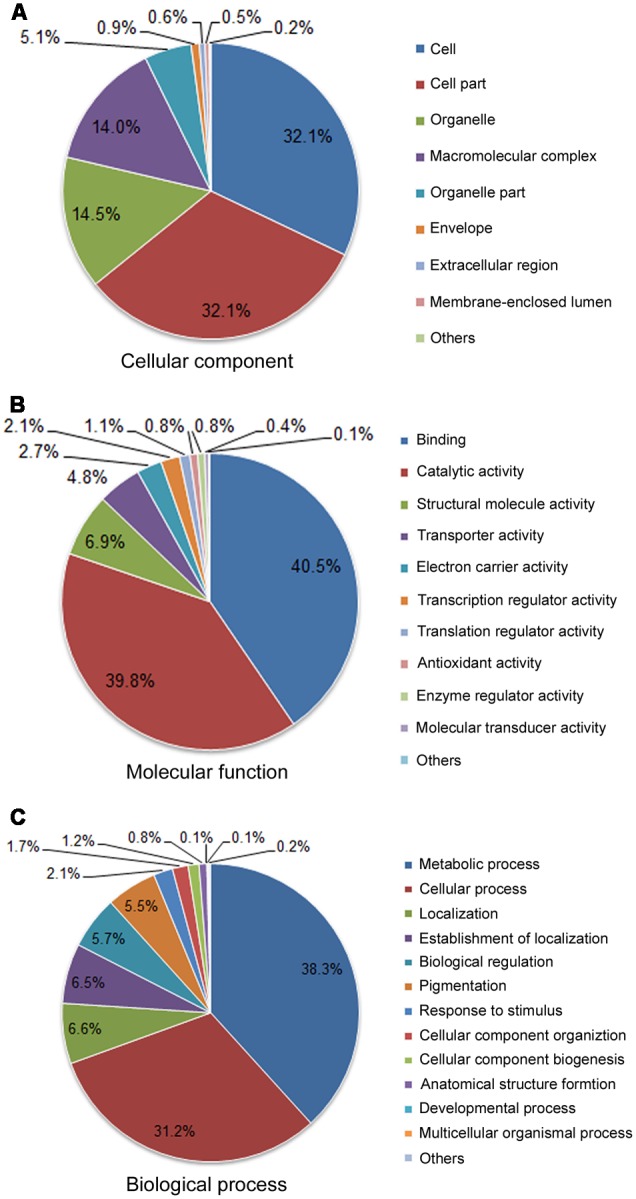
Functional classifications for the 8,835 unigenes assigned into GO terms. Pie diagrams represent the second-level GO terms for three categories: cell component **(A)**, molecular function **(B)**, and biological process **(C)**.

A total of 5,500 (62.3%) unique sequences belonged to one or more GO ontologies, of which 1,761 (19.9%), 4,934 (55.8%), and 3,858 (43.7%) unigenes were assigned the GO categories CC, MF, and BP, respectively. A total of 1,256 (14.2%) unigenes were categorized into three ontologies. The GO categories CC, MF, and BP fell predominantly into three or four subcategories (**Figure 4**). Within the CC category (second-level GO terms, **Figure 4A**), cell (32.1%) and cell part (32.1%) are most enriched, followed by organelle (14.5%) and macromolecular complex (14%). In the MF categories (**Figure 4B**), binding (40.5%) and catalytic activity (39.8%) are the most enriched; proteins having structural molecular activity (6.9%) and transporter activity (4.8%) are dramatically enriched compared to other terms.

Gene Ontology biological process is helpful for the functional classification of the analyzed genes. In the BP category (**Figure 4C**), metabolic process (38.3%) and cellular process (31.2%) accounted for the highest proportion, suggesting a high basic metabolic activity existed in the NaCl-stressed tissues; localization (6.6%), establishment of localization (6.5%), biological regulation (5.7%) and pigmentation (5.5%) were dominantly enriched; response to stimuli (2.1%) at the third level were also enriched.

### KEGG Pathway Assignment

To understand the classification of pathway mapping for *A. pumila* underlying salinity stress, we annotated unique sequences using KAAS based on molecular interaction and reaction networks. We observed that the genes were mapped to six KEGG biochemical pathways (**Table 4**), including metabolism (3,405 unigenes, 38.5%), genetic information processing (GIP, 2,224, 25.2%), environmental information processing (EIP, 482, 5.5%), cellular processes (CP, 565, 6.4%), organism system (OS, 729, 8.3%), human diseases (HD, 1,058, 12%). The largest group, for example, metabolism was represented by 3,405 unigenes, including energy metabolism (22% of metabolism), carbohydrate metabolism (21.3% of metabolism), amino acid metabolism (15.9% of metabolism), lipid metabolism (7.5% of metabolism), and biosynthesis of other secondary metabolites (4.2% of metabolism). GIP was the second largest group, with a majority of encoding proteins involved in translation (43.5% of GIP), and folding, sorting, and degradation (31.4% of GIP). In the category of EIP, signal transduction (65.8%), were the majority as oppose to signal molecules and interaction (26.7% of EIP), and membrane transport (7.5% of EIP). Transport and catabolism (49% of CP) and cell growth and death (24.6% of CP) constituted the majority of CP category. The major constitutions of OS were immune system (23.2%), endocrine system (21.1%), and environmental adaptation (16%).

**Table 4:** The distribution of KEGG pathway.

KEGG categories represented	No. of unigenes	Percent of unigenes (%)	Percent of categories (%)
**Metabolism (3,405, 38.5%)**			
Amino acid metabolism	541	6.1%	15.9%
Biosynthesis of other secondary metabolites	143	1.6%	4.2%
Carbohydrate metabolism	726	8.2%	21.3%
Energy metabolism	749	8.5%	22%
Enzyme families	258	2.9%	7.6%
Glycan biosynthesis and metabolism	111	1.3%	3.3%
Lipid metabolism	256	2.9%	7.5%
Metabolism of other amino acids	152	1.7%	4.5%
Metabolism of terpenoids and polyketides	95	1.1%	2.8%
Nucleotide metabolism	103	1.2%	3%
Other metabolism	120	1.4%	3.5%
Xenobiotics biodegradation and metabolism	151	1.7%	4.4%
**Genetic information processing (GIP, 2,224, 25.2%)**			
Folding, sorting, and degradation	698	7.9%	31.4%
Replication and repair	221	2.5%	9.9%
Transcription	337	3.8%	15.2%
Translation	968	11%	43.5%
**Environmental information processing (EIP, 482, 5.5%)**			
Membrane transport	36	0.4%	7.5%
Signal transduction	317	3.6%	65.8%
Signaling molecules and interaction	129	1.5%	26.7%
**Cellular processes (CP, 565, 6.4%)**			
Cell communication	83	0.9%	14.7%
Cell growth and death	139	1.6%	24.6%
Cell motility	66	0.7%	11.7%
Transport and catabolism	277	3.1%	49%
**Organismal systems (OS, 729, 8.3%)**			
Circulatory system	48	0.5%	6.6%
Development	32	0.4%	4.4%
Digestive system	41	0.5%	5.6%
Endocrine system	154	1.7%	21.1%
Environmental adaptation	117	1.3%	16%
Excretory system	67	0.8%	9.2%
Immune system	169	1.9%	23.2%
Nervous system	83	0.9%	11.4%
Sensory system	18	0.2%	2.5%
**Human diseases (HD, 1,058, 12%)**			
Cancers	177	2%	16.7%
Cardiovascular diseases	17	0.2%	1.6%
Immune system diseases	83	0.9%	7.8%
Infectious diseases	320	3.6%	30.3%
Metabolic diseases	21	0.2%	2%
Neurodegenerative diseases	440	5%	41.6%

### Identification of Putative Transcription Factors and Salinity Stress-Related Genes

Plant transcription factor database (TFDB 4.0) consists of 165 species covering the main lineages of the green plants (Pérez-Rodríguez et al., 2010; Jin et al., 2017). TFs identification using TFDB 4.0 predicted 251 (2.8% of unigenes) TFs that best match with *A. thaliana* (*E*-value < 10^-5^) (**Supplementary File S4**), which are classified into 42 families. The most abundant TF family was the ERF group (27 unigenes, 10.8%), followed by NAC (23, 9.2%), C2H2 (18, 7.2%), bHLH (15, 6%), bZIP (15, 6%), GRAS (12, 4.8%), G2-like (11, 4.4%), WRKY (11, 4.4%), HD-ZIP (10, 4%), MYB (9, 3.6%), and MYB related (9, 3.6%) (**Table 5**).

**Table 5:** The most abundant putative transcriptional factors (TFs).

TF family	TF description	Total of ESTs	Total of unigenes	Redundancy^a^	Percent (%)^b^
ERF	Single AP2/ERF domain	42	27	1.6	10.8%
NAC	No apical meristem (NAM) domain	43	23	1.9	9.2%
C2H2	Zinc finger, C2H2 type	27	18	1.5	7.2%
bHLH	Basic/helix-loop-helix domain	20	15	1.3	6%
bZIP	Basic leucine zipper (bZIP) motif	19	15	1.3	6%
GRAS	Three initially identified members, GAI, RGA, and SCR	15	12	1.3	4.8%
G2-like	Golden2-like (GLK)	13	11	1.2	4.4%
WRKY	WRKY RNA-binding domain	14	11	1.3	4.4%
HD-ZIP	Homeobox with a leucine zipper motif	23	10	2.3	4%
MYB	Myb-like DNA binding domain	14	9	1.6	3.6%
MYB_related	Myb related family domain	9	9	1.0	3.6%

*^a^Redundancy is (Total of ESTs)/(Total of unigenes). ^b^Percent is (Total of unigenes)/(Total of putative TFs, 257).*

During the past few years, many genes related to salt tolerance or involving in response to salt stress have been discovered in a large number of plant species, and several excellent reviews and research articles are available (Hasegawa et al., 2000; Zhu, 2002; Jha et al., 2009; Shavrukov, 2013; Gupta and Huang, 2014). According to these papers, in addition to TFs, we classified the genes that may be related to salt stress-tolerance of our high quality ESTs into main seven groups based their putative major function like osmolyte biosynthesis gene, transmembrane transport and ion homeostasis, ROS scavengers, general stress proteins, signaling components, membrane fluidity and metabolism, The genes represented in each category were listed in **Table 6**.

**Table 6:** Represented genes that may be related to salt stress-tolerance.

Class of target	Functional annotation	Unigenes name/Accession number	EST number	Matching with	*E*-value
(1) Osmolyte biosynthesis	Acid beta-fructofuranosidase 4	Contig2157	2	*A. lyrata*	0
gene	Alpha-trehalose-phosphate synthase (TPS10)	Contig2925	2	*A. thaliana*	*E* - 135
	2-alkenal reductase (AER)	Contig369	1	*A. thaliana*	0
	Betaine aldehyde dehydrogenase 2 (ALDH10A9)	Contig2945	2	*A. thaliana*	*E* - 152
	Delta-1-pyrroline 5-carboxylase synthetase 1 (P5CS1)	Contig4970	18	*A. thaliana*	0
(2) Transmembrane	Ca^2+^-transporting ATPase	Contig2566	2	*A. thaliana*	5*E* - 24
transport and	CBL-interacting serine/threonine-protein kinase 9 (SOS2)	Contig3662	3	*A. thaliana*	0
ion homeostasis	CBL-interacting serine/threonine-protein kinase 6 (SOS3)	Contig3526	3	*A. thaliana*	1*E* - 140
	H^+^-transporting ATPase	Contig3622	2	*A. thaliana*	1*E* - 90
	Multidrug resistance protein ABC transporter	Contig4793	6	*M. truncatula*	0
	Plasma membrane H^+^-ATPase 3 (PM-H^+^-ATPase)	Contig4385	4	*A. thaliana*	1*E* - 106
	Sodium/hydrogen antiporter 1 (NHX1)	JZ944758	1	*A. thaliana*	1*E* - 162
	Vacuolar-type H^+^-pumping pyrophosphatase 1 (VP1)	Contig4134	2	*A. lyrata*	1*E* - 162
	V-type proton ATPase subunit E1 (V-ATPase)	Contig4804	8	*A. thaliana*	1*E* - 121
(3) Reactive oxygen	Catalase 3 (CAT3)	Contig5024	52	*B. rapa*	0
scavengers	Glutathione *S*-transferase 16 (GSTF3)	Contig5012	31	*A. thaliana*	1*E* - 111
	L-Ascorbate peroxidase	Contig3656	2	*A. thaliana*	1*E* - 153
	Metallothionein 2B (MT2B)	Contig4911	12	*A. thaliana*	1*E* - 40
	Monodehydroascorbate reductase (NADH)	Contig4730	7	*A. thaliana*	0
	Peroxidase 42	Contig4650	6	*A. thaliana*	0
	Plasma membrane intrinsic protein 2A (PIP2A)	Contig4691	6	*A. lyrata*	0
	Thioredoxin H3 (TRXH3)	Contig4961	19	*B. divaricarpa*	1*E* - 48
(4) Stress protein	Aquaporin PIP1-2 (PIP1B)	Contig4912	12	*B. rapa*	1*E* - 163
	Cysteine proteinase RD21a (RD21a)	Contig4976	18	*A. thaliana*	0
	Dehydrin ERD14 (ERD14)	Contig4956	16	*A. thaliana*	3*E* - 84
	Heat shock protein 81-4 (HSP81-4)	Contig2465	2	*A. thaliana*	0
	Late embryogenesis abundant protein (LEA3)	Contig4296	4	*A. lyrata*	1*E* - 138
	low temperature and salt responsive protein LTI6B	JZ932940	1	*A. thaliana*	1*E* - 80
	Osmotin-like protein (ATOSM34)	Contig3081	2	*A. thaliana*	1*E* - 138
	Salt tolerance-like protein (STH)	Contig3575	2	*A. thaliana*	1*E* - 111
	Stress-induced protein KIN2 (KIN2)	Contig3436	2	*A. thaliana*	2*E* - 26
(5) Signaling components	Calcium-dependent protein kinase 13 (CDPK13)	Contig2514	2	*A. lyrata*	3*E* - 94
	Calcium-binding protein CML13 (CML13)	JZ947887	1	*A. thaliana*	3*E* - 78
	CBL-interacting serine/threonine-protein kinase 6 (CIPK6)	Contig3722	2	*A. thaliana*	1*E* - 140
	Glycine-rich protein	Contig4824	8	*A. thaliana*	1*E* - 59
	GTP-binding protein (GTPASE)	Contig3680	3	*H. annuus*	1*E* - 128
	Leucine-rich repeat family protein	Contig4870	10	*A. thaliana*	0
(6) Metabolism	Aldehyde dehydrogenase 7B4 (ALDHB74)	Contig1650	1	*A. thaliana*	1*E* - 133
	Cytosolic malate dehydrogenase	Contig4330	4	*A. lyrata*	0
	Glyceraldehyde-3-P dehydrogenase	Contig4944	15	*B*. *vulgaris*	1*E* - 171
	Phosphoenolpyruvate carboxykinase 1 (PCK1)	Contig820	1	*A. thaliana*	1*E* - 150
	Putative protein phosphatase 2C	Contig3997	3	*A. thaliana*	1*E* - 113
(7) Membrane fluidity	Bifunctional inhibitor/lipid-transfer protein	Contig4587	5	*A. lyrata*	3*E* - 86
	Lipid-transfer protein DIR1-like	JZ947168	1	*V. vinifera*	5*E* - 89
	Non-specific lipid-transfer protein 4 (LTP4)	Contig4743	4	*A. thaliana*	5*E* - 54

### qRT-PCR Analyses

**FIGURE 5:**
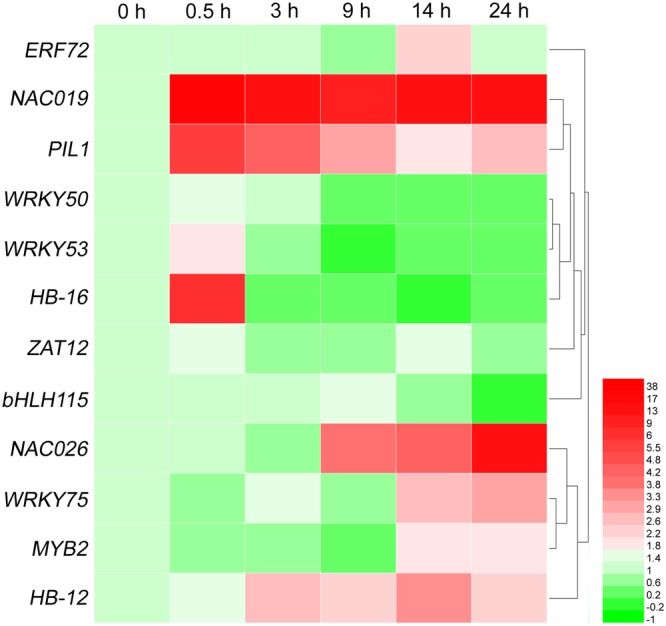
Heat-map of differentially expressed genes of 12 transcription factor families at six time points of salt shock. Each line represents a different gene. Each color represents a different gene expression level. qRT-PCR was used to evaluate the relative levels of these unigenes at six time points of 500 mM NaCl stock. The patterns were clustered and visualized by heatmap program HemI 1.0 (Deng et al., 2014).

To validate our ESTs, we first selected 12 genes from the most abundant 11 groups of TFs (**Table 5**) to determine their expression levels by qRT-PCR. The results showed that they were up- or down-regulated in response to high-salt stress, including one *ERF* (*ERF72*) gene, two *NAC* genes (*NAC019* and *NAC026*), one *C2H2* gene (*ZAT12*), two *bHLH* genes (*PIL1* and *bHLH115*), three *WRKY* genes (*WRKY50, WRKY53*, and *WRKY75*), two *HD-ZIP* genes (*HB-12* and *HB-16*), one *MYB* gene (*MYB2*). As shown in **Figure 5**, *ERF72, NAC019, PIL1, NAC026, WRKY75, MYB2*, and *HB-12* were obviously up-regulated at 14 h of 500 mM NaCl shock.

**FIGURE 6:**
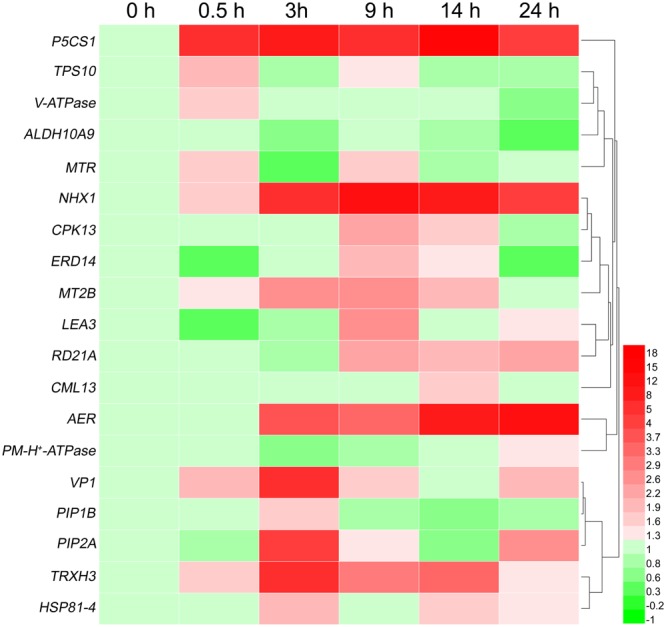
Heat-map of differentially expression of 19 select genes at six time points of salt shock. Each line represents a different gene. Each color represents a different gene expression level. qRT-PCR was used to evaluate the relative levels of the 19 unigenes at six time points of 500 mM NaCl shock. The patterns were clustered and visualized by heatmap program HemI 1.0 (Deng et al., 2014).

Furthermore, another 19 salt stress related genes from the seven groups in **Table 6** were chosen for validation. The results showed that all 19 genes were up- or down-regulated at different time of salt stress (**Figure 6**), including four osmolyte biosynthesis genes (*P5CS1, TPS10, ALDH10A9, AER*), 6 transmembrane transporters and ion homeostasis genes (*V-ATPase, PM-H^+^-ATPase, VP1, PIP2A, MTR, NHX1*), 2 reactive oxygen scavengers genes (*MT2B* and *TRXH3*), 5 stress proteins genes (*HSP81-4, LEA3, RD21A, ERD14, PIP1B*), and 2 signaling components genes (*CPK13* and *CML13*). Of the 19 salt-induced genes, *P5CS1, NHX1, AER*, and *TRXH3* were dramatically up-regulated at 14 h of salt stress.

## Discussion

### Gene Enrichment for Salt Shock Tolerance in *A. pumila*

To survive the adverse environmental conditions, such as high temperature and high salinity, ephemerals in the desert and saline-alkaline lands have evolved many distinctive adaptation mechanisms (Wang et al., 2003; Tu et al., 2016). *A. pumila*, an ephemeral plant, has a good ecological adaptability with sensitive responsiveness to environmental changes during the growth and development stages. It is not a halophyte like salt cress (*T. halophila*), nor a glycophyte like *A. thaliana*. Although it does not possess salt glands, *A. pumila* has very good salt tolerance, thus providing a good insight into the adaptive mechanisms of salt stress in the *Brassicaceae* (Xu et al., 2013; Zhao et al., 2013). EST sequencing and analysis is still an effective technology for genes annotation and discovery in modern molecular biology and genomics studies, as it provides a valuable insight into the genomic mechanisms underlying diverse environmental responses, especially for those species where genomic information is unavailable (Zhou et al., 2016).

In the present study, 16,014 high-quality ESTs from the *A. pumila* cDNA library were generated and assembled into 8,835 unigenes. 8,011 unigenes showed similarity with known proteins, while 425 unidentified unigenes could be regarded as novel genes unique to *A. pumila*, whose functions would need to be studied further.

Further analyses showed that a large number of ESTs were related to biotic and abiotic stimulus responses according to the GO biological process category (**Figure 4A**) and function annotations (**Table 6**). Many previously reported salt-related genes were also manifested in this library, for example *VP1, NHX1, SOS2, SOS3, NAC, MYB, ERF, LEA, P5CS1*, and others. The identification of putative salt stress-related genes in the dataset would be helpful in revealing the adaptation mechanisms for *A. pumila* in response to environmental stimuli.

The openly available ESTs sequences obtained provide a resource for salt-tolerance gene mining and molecular marker identification related to growth and development in *A. pumila*.

### Identification of Genes Responsible for Salt-Response and Transcriptional Regulation of Salt Stress Response in *A. pumila*

The NaCl shock conditions applied to the plants used in the preparation of the cDNA library are effective in inducing the typical salt-tolerance process not only in halophytes but also in non-halophyte (Shavrukov, 2013), and the results can therefore be considered to be a general strategy to identify ESTs related to salt stress response. Not only were large numbers of transcripts related to photosynthesis identified in the present cDNA library (**Table 2** and **Supplementary File S2**), but so were genes related to salt stress (**Table 6**), indicating that the salt shock treatment might suppress the process of photosynthesis and induce the salt stress defenses in *A. pumila*.

High levels of salinity (200–500 mM) can cause osmotic shock and plasmolysis in plant root cells, so osmotic and ionic stresses are generally regarded to be the two main components of salt stress or shock (Shavrukov, 2013). After exposure to salt stress, however, plants can achieve osmotic homeostasis by adjusting osmotically for water potential and turgor (Munns, 2002). Cell turgor maintenance, accumulation of soluble sugars and other osmolytes, and water balance are the most important physiological processes of osmotic adjustment, and they are controlled by genes with osmotic function (Munns, 2005). Many genes identified are directly related to osmotic shock responses, for example *P5CS1* and *ALDH10A9*, (**Figure 6** and **Table 6**). Many ESTs related to proline synthesis were also found in the EST database. Under high salinity shock conditions, the expression of *P5CS1* (Contig4970), encoding delta-1-pyrroline 5-carboxylase, a bifunctional enzyme for proline biosynthesis, was induced early at 0.5 h, continued to increase over the 24 h period (**Figure 6**).

Expression of betaine aldehyde dehydrogenase gene *ALDH10A9* (Contig2945), which is functionally involved in the synthesis of the osmolyte glycine betaine, was also up-regulated during response to salt shock. Many ESTs related to sugar synthesis were present in the library. Previous study had found that trehalose functions as an osmoprotectant and prevents abiotic stress from damaging organisms (Chary et al., 2008). Trehalose-6-phosphate synthase (TPS) is involved in the first step of trehalose synthesis and plays an important role in trehalose synthesis (Jiang et al., 2010). The expression of *TPS10* (Contig2925), encoding a trehalose biosynthesis enzyme, was up-regulated at 0.5 h and 9 h of salinity shock. In *A. thaliana*, an alkenal reductase (AER) homolog was possibly involved in NAD/NADH homeostasis and played a role in antioxidant defense, but its molecular mechanism remains unclear (Babiychuk et al., 1995). Expression of an *A. pumila AER* gene was dramatically up-regulated at 3 h, and continued to be highly expressed in response to salinity stress, suggesting a distinct role in salinity tolerance (**Figure 6**).

If exposed to salt for a long time, plants can also restore ionic, to achieve greater tolerance, by transferring excess Na^+^ from the cytoplasm into the apoplast, and sequestering of Na^+^ from the cytosol to the vacuole by using specific plasma membrane and vacuole sodium/hydrogen antiporters (Na^+^/H^+^ antiporter, NHX1). It is well documented that the plasma membrane and tonoplast NHX (Apse et al., 1999; Gaxiola et al., 2001), the plasma membrane H^+^-adenosine triphosphatase (PM-H^+^-ATPase), the tonoplast H^+^-ATPase (V-ATPase) and the H^+^-inorganic pyrophosphatase (V-H^+^-PPase, VP) (Maeshima, 2001) regulate sodium ion concentration coordinately. Secondary active transport and electrochemical flux across the plasma membrane and tonoplast are driven by a H^+^ pump which is powered by PM-H^+^-ATPase, V-ATPase, and VP (Maeshima, 2001; Jha et al., 2009).

In the unique ESTs of *A. pumila*, a vacuolar Na^+^/H^+^ antiporter gene *NHX1* (JZ944758), CBL-interacting serine/threonine-protein kinase 9 (*SOS2*, Contig3662), CBL-interacting serine/threonine-protein kinase 6 (*SOS3*, Contig3526), multidrug resistance protein ABC transporter family (Contig4793) were present; *PM-H^+^-ATPase* (Contig4385), *V-ATPase* (Contig4804), *VP1* (Contig4134) and several related *ATPase* (Contig2566 and Contig3622) were also present (**Table 6**). In *A. pumila, NHX1* and *VP1* expression were significantly up-regulated under high salt conditions, and the highest expression of *NHX1* was observed at 9 h, whereas for *VP1*, it was 3 h and for *V-ATPase* it was 0.5 h, respectively (**Figure 6**). However, *PM-H^+^-ATPase* expression was only up-regulated after 0.5 and 24 h of NaCl treatment, and even down-regulated at 3 and 9 h. Thus, PM-H^+^-ATPase may be less important physiologically than VP in *A*. *pumila* under salt shock. Although, >16,000 cDNA clones were sequenced, some genes may be expressed at too low a level to be identified. The plasma membrane-localized Na^+^/H^+^ antiporter SOS1 (Zhu, 2000) and sodium transporter HKT1 (Rubio et al., 1995) have been demonstrated to play an essential role in salt tolerance, but unigenes encoding these proteins were not discovered in this study. The exact reason for this discrepancy needs further study.

In addition, water channel proteins such as plasma membrane intrinsic protein 2A (PIP2A, Contig4691) might be involved in controlling the speed of water flux across cellular membranes under salt stress to help sustain osmotic homeostasis (Zhu, 2001). qRT-PCR results showed that *PIP2A* expression was up-regulated at 3, 9, and 24 h of salt treatment, with the highest expression being at 3 h.

Salinity stress leads to increases in amount of ROS such as singlet oxygen, peroxides, superoxide, hydroxyl radical, and hydrogen peroxide (Hasegawa et al., 2000; Jha et al., 2009; Gupta and Huang, 2014), resulting in significant damage to the cell structure. Plants produce different antioxidant enzymes, which can scavenge free radicals, such as catalase (CAT), sodium dismutase (SOD), ascorbate peroxidase (APX), GST and glutathione reductase (GSR). Many genes related to ROS scavenging were present in this database. Among the 8,835 unigenes, *CAT3* (Contig5024) contained 53 ESTs, *GSTF3* (Contig5012) contained 37 ESTs and *GST1* (Contig4958) contained 15 ESTs (**Table 2**). These genes were also reported in the EST database of the halophytes *Thellungiella salsuginea* (Wong et al., 2005) and *Salicornia brachiata* (Jha et al., 2009), as well as rice (Kawasaki et al., 2001). qRT-PCR analysis showed that the expression in *A. pumila* of both *CAT3* and *GSTF3* were significantly induced at 14 h of salinity shock (**Figure 2**).

Metallothioneins (MT) are a group of cysteine-rich, low-molecular-weight metal-binding proteins that are thought to be involved in metal ion metabolism and detoxification (Hall, 2002). MT-like transcripts have been reported to be highly up-regulated in response to salt stress in barley (Ozturk et al., 2002), *Citrus sinensis* (Bausher et al., 2003), and the wild soybean *Glycine soja* (Ji et al., 2006). The *MTP-2B* transcript (Contig4911) was up-regulated after 0.5 h of salt shock (**Figure 6**).

In plants, thioredoxin plays a wide range of roles in many important biological processes, ranging from photosynthesis to growth, flowering and the development and germination of seeds, as well as cell-to-cell communication (Meng et al., 2010). It is also involved in cell redox homeostasis and cellular response to oxidative stress, among others. The expression of a *TRX3* (Contig4961) gene in *A. pumila* was rapidly up-regulated in response to salinity shock, and peaked at 3 h.

Under salt shock or stress conditions, many stress proteins were also produced to enhance salt tolerance (Jha et al., 2009; Shavrukov, 2013). It was observed among the unique ESTs resources that cysteine proteinase RD21a (Contig4976), stress-induced protein KIN2 (Contig3436), late embryogenesis abundant (LEA) protein (Contig4296), dehydrin-related protein (Contig4956), aquaporin PIP1-2 (PIP1B) (Contig4912), heat shock protein 81-4 (Contig2465) and other stress-induced protein were present (**Table 6**). Analyzing gene expression time-courses in response to high salinity shock revealed that *RD21a* transcription peaked at 14 h, and LEA peaked at 9 h, whereas *PIP1B* and *HSP81-4* transcription peaked at 3 h (**Figure 6**).

Calmodulin (CaM) is an intracellular target of the secondary messenger Ca^2+^, which is involved in stress signal transduction as reported by a number of authors (Zhu, 2000; Ji et al., 2006). Once bound to Ca^2+^, CaM acts as part of a calcium signal transduction pathway by modifying its interactions with various target proteins such as kinases or phosphatases (Chin and Means, 2000). The expression of a *calmodulin*-like 13 (*CML13*, JZ947887) was slightly up-regulated as early as 0.5 h of salt treatment, peaked at 14 h and then returned normal levels at 24 h (**Figure 6**).

Calcium-dependent protein kinase (CPK) is involved in the strengthening of cell walls and in early stages of signal transduction, and it is a component of the immediate response to osmotic shock/plasmolysis (Shavrukov, 2013). Overexpression of a rice *CPK* gene has been shown to increase tolerance to low temperature, drought, and high salt in rice (Kawasaki et al., 2001). The *CPK13* (Contig2514) gene was highly expressed after 9 h of salt treatment but returned to normal levels after 24 h. Altogether, time-course analyses of the expression of the genes studied confirm that salinity tolerance phenomena in *A. pumila* involve sequential events starting with the expression of salt-responsive genes, the primary induction of key genes, followed by expression of other genes.

Transcriptional factors regulate gene expression at the transcriptional level either by binding to a specific *cis*-acting DNA sequence common to the enhancer regions of specific functionally related genes (specific TFs) or by forming a large transcription preinitiation complex that interacts with RNA polymerase directly in all genes (general TFs) (Thomas and Chiang, 2006). In plants, certain TFs can confer resistance to various stresses through up-regulation of a wide array of genes in response to environment stimuli. Approximately 5.9% of the *Arabidopsis* genome encodes transcription factors(Riechmann et al., 2000), whereas the halophyte *T. salsuginea*, only dedicates 1% of its genome to code for transcription factors or signal transduction elements (Wong et al., 2005).

In the current study, 251 putative TFs (representing 2.8% of unigenes) were identified from *A. pumila* (**Supplementary File S4**). Among these, high-frequency of TF families included ERF, NAC, C2H2, bHLH, bZIP, GRAS, G2-like, WRKY, HD-ZIP, and MYB (**Table 5**). Transcript levels of *ERF72, PIL1, NAC019, WRKY53, HB-16, NAC026, WRKY75, MYB2*, and *HB-12* were significantly up-regulated in response to salinity shock (**Figure 5**).

*NAC* gene family is one of the largest plant-specific transcription factor families. Increasing evidence indicates that NAC domain-containing proteins are also involved in plant response to biotic and abiotic stresses. In *A. thaliana, ANAC019, ANAC055*, and *ANAC072* were markedly up-regulated by drought, salt, and ABA treatment, and consequently improved plant drought resistance (Tran et al., 2004). Similarly, in the present study, *NAC019* and *NAC026* were highly expressed in response to salt stress (**Figure 5**).

bHLHs, which represent the second-largest type of transcription factor in *A. thaliana*, can regulate plant responses to multiple abiotic stresses (Heim et al., 2003). As found in this study, *A*. *pumila bHLH15* (Contig280) is homologous to *AtHLH15*, which responds to salt stress (Liu et al., 2014). The WRKY TF is primarily specific to plants and algae, and is a class of TF that plays a major role in plant defense and response to biotic stresses (Bakshi and Oelmüller, 2014). Over-expression of *GmWRKY54* enhanced drought and salt tolerance in transgenic soybean plants (Zhou et al., 2008). *A. pumila WRKY75* (Contig4437) was markedly up-regulated at 14 h of NaCl treatment (**Figure 5**).

MYB TFs comprise one of the largest TF families and are involved in controlling various processes, including biotic and abiotic responses. AtMYB2 possesses an R2R3 MYB DNA-binding domain and is known to regulate the expression of salt- and dehydration-responsive genes (Abe et al., 2003). *A. pumila MYB2* (Contig4149) was clearly up-regulated at 14 h of salt shock. Taken together, these results indicated that TFs may contribute to the regulation of salt response in *A. pumila*, and lay the foundations for further investigations into the functions of TFs in *A. pumila* under salt stress.

The database (16,014 ESTs and 8,835 unique sequences) obtained from *A. pumila* is a valuable resource, which will facilitate the comparative genomics study of salt stress response among green plants.

## Author Contributions

XH conceived and designed the experiments and performed the cDNA clones sequencing experiments. LY analyzed the ESTs data. YJ performed the gene expression experiments. JL and FL help to analyze the ESTs data. XH wrote the manuscript. All the authors discussed the results and contributed to the manuscripts.

## Conflict of Interest Statement

The authors declare that the research was conducted in the absence of any commercial or financial relationships that could be construed as a potential conflict of interest.

## Footnotes

1 http://www.ncbi.nlm.nih.gov/VecScreen/VecScreen.html

2 http://www.codoncode.com

3 http://www.ncbi.nlm.nih.gov/orffinder

4 http://www.expasy.org/sprot/

5 http://www.ncbi.nlm.nih.gov/COG/

6 http://www.genome.jp/kegg/kaas/

7 http://planttfdb.cbi.pku.edu.cn

## Supplementary Material

FILE S1 Primers used in qRT-PCR analysis.

FILE S2 The most abundant ESTs in 16014 unigenes.

FILE S3 Summary of ORF and amino acids.

FILE S4 List and categories of putative transcription factors.
